# Health Information Economy: Literature Review

**DOI:** 10.5539/gjhs.v7n6p250

**Published:** 2015-04-16

**Authors:** Kamal Ebrahimi, Masoud Roudbari, Farahnaz Sadoughi

**Affiliations:** 1 School of Health Management and Information Science, Iran University of Medical Science, Tehran, Iran; 2 School of Public Health, Iran University of Medical sciences, Tehran, Iran

**Keywords:** information science, health information economy, health information exchange, health information management, consumer health information consumer, health information technology

## Abstract

**Introduction:**

Health Information Economy (HIE) is one of the broader, more complex, and challenging and yet important topics in the field of health science that requires the identification of its dimensions for planning and policy making. The aim of this study was to determine HIE concept dimensions.

**Methods:**

This paper presents a systematic methodology for analyzing the trends of HIE. For this purpose, the main keywords of this area were identified and searched in the databases and from among 4775 retrieved sources, 12 sources were studied in the field of HIE.

**Results:**

Information Economy (IE) in the world has passed behind four paradigms that involve the information evaluation perspective, the information technology perspective, the asymmetric information perspective and information value perspective. In this research, the fourth perspective in the HIE was analyzed. The main findings of this research were categorized in three major groups, including the flow of information process in the field of health (production. collection, processing and dissemination), and information applications in the same field (education, research, health industry, policy, legislation, and decision-making) and the underlying fields.

**Conclusion:**

According to the findings, HIE has already developed a theoretical and conceptual gap that due to its importance in the next decade would be one of the research approaches to health science.

## 1. Introduction

Stiglitz declared that in the field of economics, perhaps the most important break with the past lies in the Information Economy (IE) theories (Stiglitz, 2000). IE is one of the broad, complex, challenging and yet very important concepts (Shapiro & Varian, 2013; Barman, 2006), that requires its dimensions to be identified for planning and policymaking. IE issues are even more complicated by the advent of information and communication technology (Biswas, 2004; Castells, 2011). In addition, the issue of Health Information Economy (HIE), due to its data and clinical information, is considered more complicated than other fields of IE (Mandl & Kohane, 1994).

In various literature, the IE is expressed as one of the basic needs for economic development (UNCTAD, 2012), quality improvement of health services, problems of asymmetric information in the health field (Haas-Wilson, 2001), cost reduction of resources, information services (Coiera, 2001), and information security improvement (Langenderfer & Miyazaki, 2009). Wanger believes that the IE perspective leads to a change in thinking about the flow of information in the health care fields (Wanger, 1999). More and Martin also name it in line with the evolution of the health service (More & Martin, 1998), and Mandl and Kohane knows it as a great change in the health field (Mandl & Kohane, 2009).

IE has passed behind different paradigms that can be divided in four approaches. These approaches are information evaluation, information technology evaluation, asymmetric information, and information value perspective.

The first approach of IE theory is based on information sector evaluation. Machlup and Port performed two main studies in this field. Their famous studies determine the flow of movement from an industrial society to an information society, and try to show the characteristics of the information society (Godwin, 2008). Porat added a new section called information to the traditional sectors of the economy (agriculture, industry and services), introduced a fundamental change in the economic structure. From Porat’s perspective, this section is divided into the main and secondary sectors. The product of the main section is information, while in the second section, products and services are manufactured which are based on information (Porat, 1977).

The second paradigm in IE, is information technology economy, that can be observed in the reports of the next decades of the Organization for Economic Co-operation and Development (OECD) and activities of United Nations Conference in Trade And Development (UNCTAD). In fact, the IE has the approach of information technology. UNCTAD’s annual reports from 2005 to 2012 are in this aspect (UNCTAD, 2012; OECD, 2012).

The third paradigm is a conceptual approach. This approach is Information asymmetry. Information asymmetry deals with the study of decision in transaction where one party has more or better information than the other. Akerlof demonstrated how a market can collapse if critical pieces of information are missing. In 2001 the Nobel Prize in Economics was awarded to George Akerlof, Michael Spence, and Joseph E. Stiglitz for their analyses of markets with asymmetric information (Akerlof, Spence, & Stiglitz, 2001; Stiglitz, 2002). In addition, this issue has been discussed in the health care literature (Cardon & Hendel, 2001).

The other type of conceptualization of the IE to emerge focuses on the information value (Godin, 2008). Shapiro & Varian discuss the value of information as a commodity or new economy approach (Shapiro & Varian, 2001). In this approach, information and data are core concepts.

This can give rise to a new approach in the IE. From the perspective of information value, the overflow of information and access to information on the current location plays an important role in marketing information. The approach to information as a commodity is considered independent and different from other goods (Shapiro, Varian, & Becker, 1999; Shapiro & Varian, 2013).

This discussion illustrates the magnitude, complexity and, importance of the IE and at the same time indicates that the next decade will be the decade of IE research and policy age. The HIE is also more complex topics due to the health information and clinical data. Despite of HIE importance, research showed the lack of theory criticism in this field. The aim of this study was to evaluate different views on the economy of the world’s health information in order to determine the dimensions of the field. A systematic review was undertaken in order to provide understanding of the global status of HIE theoretical dimensions.

## 2. Methods

In This paper, a systematic review was used of the HIE literature, using a structure strategy in English and Persian language from 1964 to 2014. Search strategies were performed the following databases:

Scopus, Web Of Science, Pubmed, Science Direct, Doaj, Ebsco, OCLC, Google Scholar, Yahoo, Eric, Google books, Proquest, Jstore.

Major search keywords consist of: HIE, health information economics, health economy of information, health economics of information and medical data economy.

Publications selected based on the inclusion /exclusion criteria.

Inclusion criteria includes criteria pertaining to publication characteristics, such as full text publication, peer reviewed publication, English and Persian language publication.

Exclusion criteria includes duplicate publications, asymmetric information and research with a focus on information and communication technology removed.

**Figure 1:**
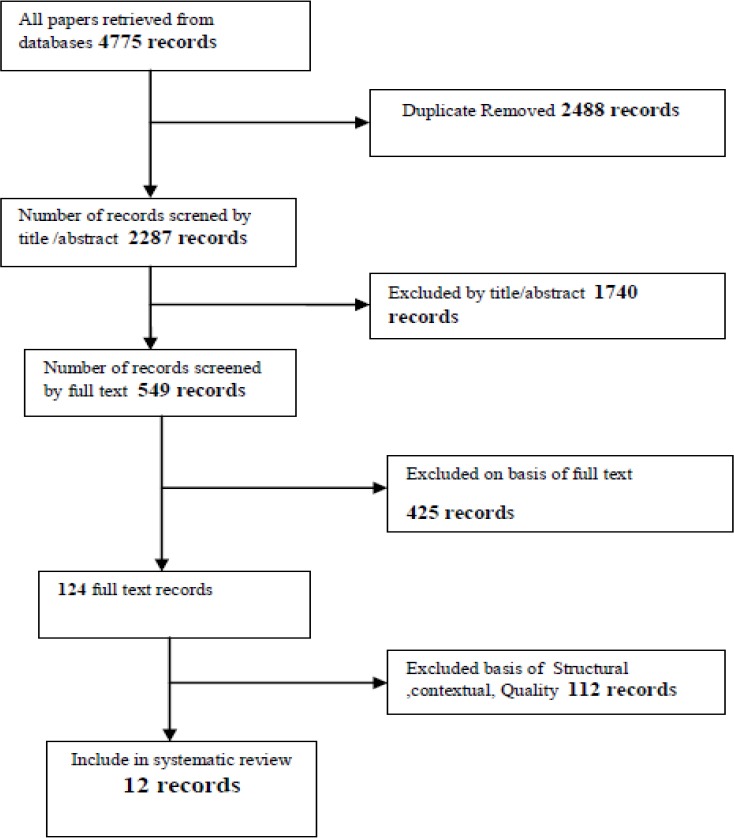
Literature flow diagram of inclusion/exclusion criteria

The inclusion/exclusion results of the search are shown in Figure 1.

## 3. Results

The databases search yield, 4775 papers. However, review of the title excluded 2488 duplicated studies. After reviewing abstract and full text studies, in total 12 studies met the inclusion criteria. The selected studies information is presented in Table 1.

**Table 1:** Health Information Economy (HIE) selected literature

Author(s)		Title	Document Type	Year	Citation
1	Olsen	The Economics of Information: Bibliography and Commentary on the Literature	Article	1971	15
2	Porat	The information economy: definition and measurement	Book	1977	1800
3	Mandl & Kohane	Tectonic shifts in the health information economy	Journal	1994	138
4	More & Martin	Quantitative health research in an emerging information economy	Journal	1998	5
5	Wagner	The economics of consumer health information: a microeconomic analysis of the use and effects of providing self-care information to a community	Dissertation	1999	
6	Coiera	Information economic and the internet	Journal	2000	62
7	Haas-Wilson	Arrow and the information market failure in health care: the changing content and sources of health care information	Journal	2001	50
8	Coiera	Maximising the uptake of evidence into clinical Practice An information economics approach	Journal	2001	20
9	Karmarkar & Apte,	Operations management in the information economy: Information products, processes, and chains	Journal	2007	83
10	Asefzadeh, Rezapour & Ghodousinezhad,	Cost Analysis of Information Generation in Alborz Healthcare(Iran)	Journal	2010	
11	Rinaldi, Capello, & Gaddi	Medical Data, Information Economy & Federative Networks: The Concepts Underlying the Comprehensive Electronic Clinical Record Framework	Book	2013	7
12	Soleymani	the Economics of Information Goods and Services with Emphasis on Health Field	Journal	2014	

The main subjects of HIE categorized in seven major groups expressed in Table 2.

**Table 2:** Health Information Economy (HIE) subjects in selected literature

Author		Production	Gathering	Processing	Distribution	Usage	Contextual	Attribute
1	Olsen	*	*	*	*	*	*	
2	Porat	*	*	*	*	*		*
3	Mandl & Kohane		*	*	*	*	*	*
4	More & Martin		*	*	*	*	*	
5	Wagner	*	*	*	*	*		
6	Coiera	*	*			*	*	*
7	Haas-Wilson				*	*	*	*
8	Coiera	*			*	*	*	*
9	Karmarkar & Apte			*	*	*	*	
10	Asefzadeh, Rezapour & Ghodousinezhad	*	*		*	*		
11	Rinaldi, Capello, & Gaddi	*	*	*	*	*	*	
12	Soleymani		*	*	*		*	*

In this study, the results were presented in seven categories:

The First, production of information, the Second, data collection, third, data processing, fourth information dissemination or distribution, fifth, applications of information (usage), sixth, information economy contextual aspects, and finally, information attributes are selected groups.

Health data and information applications consist of education, research, health industry, policy, legislation, and decision-making (especially proper selection of the markets).

HIE contextual aspects underlay factors such as security, privacy, copyright, and the role of technology.

Finally, the end of this research is to study the characteristics of the information as an economic commodity.

Dimensions of HIE have controversial approaches in selected studies. Therefore, all of the seven topics in HIE aspects demonstrated in conclusion part based on systematic review studies.

## 4. Discussion

In this study, Major topics of HIE categorized in seven groups. The first step is the production of information. This aspect mentioned in many studies (Porat, 1977; Wanger, 1999; Coiera 2001; Asefzadeh, Rezapour, & Ghodusinezad, 2010). The term information commodities, as used to define sectors by Porat, include products used to process or transmit information but information products in some studies mean packaged pure information (Karmarkar & Apte, 2007; Shapiro & Varian, 2013). However, the reality is that less emphasis in the areas of HIE literature devotes to information production. But Information production is important aspect in science and knowledge economy (Partha & David, 1994; Diamond, 1996; Stephan, 1996).

Nowadays information is available so quickly, and so expensively and broadly. The problem is not information production or information access but information overload. Nobel Prize winner economist Herbert Simon believes that “A wealth of information creates a poverty of attention” (Coiera, 2001; Shapiro & Varian, 2001).

The second dimension in the HIE is information collection. More and Martin points that organizations have been forced into better information collection and management strategies in order to secure their positions in the internal market. Increasingly, government agencies face pressures for cost recovery in the collection and analysis of large datasets (More & Martin, 1998). Also information interoperability and information exchange problems relate to health data and information gathering (More & Martin, 1998; Mandl & Kohan, 1994).

The third dimension is information processing that transform inputs into outputs. Information processing activities emphasize to added value of information (Mandl & Kohane, 2009).

Taylor provides a useful schema for the information life cycle as it moves from data to information and knowledge. Taylor goes on to describe elements of the processes by which data gets moved to action including classifying, relating, formatting, signaling, displaying selecting, analyzing, validating, comparing, and interpreting (Taylor, 1982; Wheeler, 2011).

Shapiro and Varian pointed that the real value produced an information provider comes in locating, filtering and communicating (Shapiro & Varian, 2013). Wenger also believes that the relationship between customer and information supplier will help to improve the quality of information (Wanger, 1999).

The fourth dimension is information dissemination. The dissemination of data is now seen as an important aspect of revenue generation (More & Martin, 1998). The first approach to the provision of information in the economics of health information and health services market refers to the flow of information (Bates & Benjamin, 1990; Has-Wilson, 1997).

The technology makes information more accessible and more valuable. Biswas declared that web will influence the traditional information economy research (Biswas, 2004). Web continues to grow, offering seemingly unlimited potential for the creation, storage, and dissemination of information (Coiera, 2000; Biswas, 2004).

Pricing, costs, and benefits associated with complex electronic databases, web development, and information technology are their current concern and this topics can be seen as another aspects of the information economy (Wheeler, 2011).

Stiglitz declared that economic cost of commodities that the consumer pays the price, and search for it (Stiglitz, 2000). However, if the cost of access to information on the internet is free but not eliminate transaction costs and searching costs.

In addition, Coiera review showed that a basic economic analysis of the current growth of information on the Internet has substantial implications for information retrieval by consumers (Coiera, 2000).

In the area of health, access to evidence-based information via valid databases is important for reducing the adverse effects of invalid data in the medical field (Gostin, 2001; Coiera, 2001).

The value of the web and valid databases lies in its capacity to provide immediate access to information and improve ability to manipulate information. New technologies aid to create a collaborative environment in health context, in which the actors belonging to different organizations and roles can collaborative sharing data and information to improve health information services (Rinaldi, Capello, & Gaddi, 2013).

The fifth dimension is information applications (usage). Health data and information applications consist of education, research, health industry, policy, legislation, and decision-making (especially proper selection of the markets) (More & Martin, 1998; Coiera, 2001; Rinaldi et al., 2013).

Information applications are the oldest and comprehensive aspect in information economy literature (Bates, 1999; Olsen, 1971; Mandl & Kohane, 2009).

Health Information systems and health information technology economy are the other related topics in information applications (O’Reilly et al., 2012; Buntin, 2011; Petit, 2001).

The sixth dimension is information economy underlying aspects. Privacy and security are fundamental topics in this dimension. Gostin believes that the success of the health care system (Also HIE) depends on the accuracy, correctness and trustworthiness of the information, and the privacy and security to control the disclosure of personal information (Gostin, 2001).

In many studies (Reidenberg, 1991; Langenderfer & Miyazaki, 2009; Fairchild et al., 2007) privacy and security importance in health information activities mentioned.

Finally, information attributes is part of HIE topics. Information is unusual commodity so IE has especial aspects different from other commodities and requires new rules for itself and the first step is to recognize the characteristics of the information commodity (Shapiro & Varian, 2013; Barman, 2006). In addition, planning IE depends on understanding the characteristics of information commodities.

Varian mentioned that information has three main properties that would seem to censuses problems for market transaction. It is experience commodity and you must experience an information commodity before you know what it is. Information typically has a high fixed cost of production but a low marginal cost of reproduction and information commodities also typically non-rival and sometimes non excludable (Shapiro & Varian, 2013). Braman provides a detailed exposition of the nature of information. In these categories, information is different from other commodities: Information is intangible with no set unit of measure and very heterogeneous (Braman, 2006). Information and data can be changed without changing the information media. Intellectual property can be produced with minimal dependence on physical property and regenerate very low cost, fast and easy (Soleymani, 2014; Godin, 2008).

## 5. Conclusion

IE has passed different paradigms that can be divided in four approaches. These approaches are information evaluation, information technology evaluation, asymmetric information, and information value perspective. Information value is one of the important aspects in IE theories and is one of the broad, complex, challenging and yet very important concepts. In addition, the issue of HIE, due to its data and clinical information, is considered more complicated than other fields of IE. Therefore dimensions of HIE have controversial approaches in selected studies.

production of information, data collection, data processing, information dissemination or distribution, applications of information (usage), information unusual attributes as a commodity, and security and safety are major controversial dimension in HIE.

We believe that HIE has already developed a theoretical and conceptual gap that due to its importance in the next decade would be one of the important research approaches to health science.
